# Novel method of atrial mechanical sensing for leadless atrioventricular synchronous pacing

**DOI:** 10.1016/j.hrcr.2022.09.002

**Published:** 2022-09-09

**Authors:** Shuichiro Kazawa, Kazuhiro Satomi, Chifumi Kazawa, Hidetaka Murakami, Yoshinao Yazaki, Nobuhiro Tanaka

**Affiliations:** ∗ Department of Cardiology, Tokyo Medical University Hachioji Medical Center, Tokyo, Japan; † Department of Cardiology, Tokyo Medical University Hospital, Tokyo, Japan

**Keywords:** Micra AV, Leadless pacemaker, Atrioventricular block, Atrioventricular synchronous pacing, Atrial sensing

## Introduction

Leadless pacemakers (LPMs) are an alternative to conventional transvenous pacemakers. LPMs are designed to reduce the risk of complications, such as device infection and lead failure, compared to transvenous pacemakers.^1^ However, the first-generation LPMs only provide ventricular-only pacing. Recently, a new LPM (Micra AV; Medtronic, Inc, Minneapolis, MN) that enables atrioventricular (AV) synchronous pacing was approved in several countries. Micra AV has a 3-axis accelerometer sensor that provides mechanical sensing signals instead of electrical signals, facilitating AV synchronous pacing. Micra AV senses the atrial mechanical sensing signal, called the A4 signal, which is related to active atrial contraction during the ventricular diastolic phase. The A3 signal corresponds to passive ventricular filling, while the A7 signal is the fusion of A3 and A4 signals, which can be observed at a higher heart rate.^2^ However, since the A7 signal is not usually shown in the device data, the A3 threshold has to be lowered sufficiently to detect the A7 signal, but kept adequately large enough to remain above the A3 value to avoid inappropriately sensing and tracking A3 signals.

The MARVEL 2 study showed the feasibility and safety of this mechanical sensing.^3^ In this study, 95% of patients had >70% AV synchrony at rest.^3^ However, 2 previous retrospective studies about Micra AV^4^^,^^5^ demonstrated that only 65% of the patients had >70% AV synchrony, especially during a higher heart rate. These studies showed the importance of appropriate patient selection for the Micra AV and the importance of active device programming.

We experienced a patient who obtained a higher AV synchrony rate by a simplified method, indicating that atrial mechanical sensing can be achieved within an intentionally prolonged A3 window regardless of heart rate.

## Case report

The patient was an 82-year-old man with no significant past medical or family history. He was transferred to our hospital after experiencing fatigue while playing golf. At admission, his blood pressure was 80/60 mm Hg and heart rate was 35 beats per minute (bpm). A 12-lead electrocardiogram showed complete AV block. The underlying atrial rhythm was sinus at a rate of 67 bpm, and the intrinsic ventricular rate was less than 30 bpm. Transthoracic echocardiography demonstrated preserved left ventricular ejection fraction and no structural heart diseases. Mitral inflow velocity showed a relaxation disorder pattern of 0.86 of the E/A wave ratio. According to patient’s preference, we decided to implant a Micra AV after obtaining the patient’s consent. The Micra AV was successfully placed by a standard transfemoral procedure with the appropriate pacing and sensing thresholds for ventricular pacing.

### Device performance after implantation

After the implantation, the atrial sensing setup was programmed and the VDD mode was activated. One hour after the procedure, the manual atrial mechanical test was performed, which, however, showed inadequate AV synchrony, and the percentage of atrial mechanical sensing–ventricular pacing (AMVp%) was only 47.1%. A histogram showed that the low AMVp% and high percentage of ventricular pacing was probably due to sinus bradycardia, which was caused by a sedated condition. Subsequently, atrial sensing was set up as follows: (1) atrial sensing vector: 1+2; (2) sensed AV delay: 20 ms and post–ventricular atrial blanking period: 550 ms; and (3) A3 threshold, A3 window end, and A4 threshold: 3.8 m/s^2^, 800 ms, and 1.3 m/s^2^, respectively.

**Figure 1:**
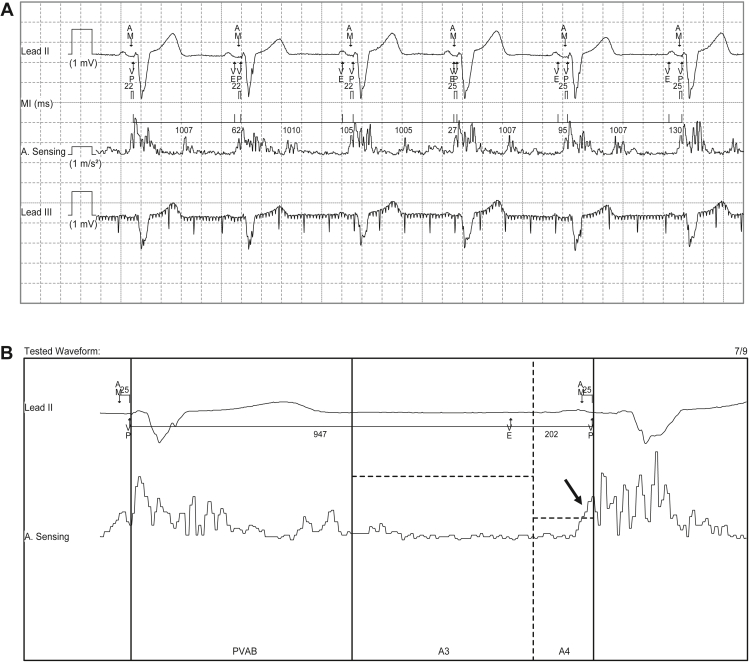
**A:** Unusual atrial mechanical sensing within the A3 window after implantation of a leadless pacemaker in this case. The figure shows a surface electrocardiogram (leads II and III) and a mechanical sensing signal of the atria after the implantation. Interestingly, no VE marker was observed, although atrial mechanical sensing and synchronous pacing were performed in the first beat, suggesting sensing of the atrial signal in the A3 window. All the subsequent beats showed VE markers, indicating that atrial signals were normally sensed in A4 windows. **B:** A manual atrial mechanical test of conventional A4 sensing The A4 signal was correctly sensed in the A4 window (*arrow*) following the end of the A3 window (= VE). AM = atrial mechanical signal; VE = ventricular end (= end of the A3 window); VP = ventricular pacing.

A device check including the manual atrial mechanical test was conducted again. Interestingly, observation of the test results showed that when the heart rate was increasing, although AV synchronous pacing could be performed, different marker patterns were observed. One was a ventricular end (VE) marker and another was a no-VE marker, both of which occurred during AV synchronous pacing (Figure 1A). The VE signal was defined at the end of ventricular contraction, indicating the end of the A3 window. The A4 signal was correctly sensed in the A4 window following the end of the A3 window (= VE) (Figure 1B). AV synchronous pacing without a VE signal suggested that the A4 signal was occasionally sensed in the A3 window with an adequate A3 threshold. This was usually observed at a moderate heart rate.

**Figure 2:**
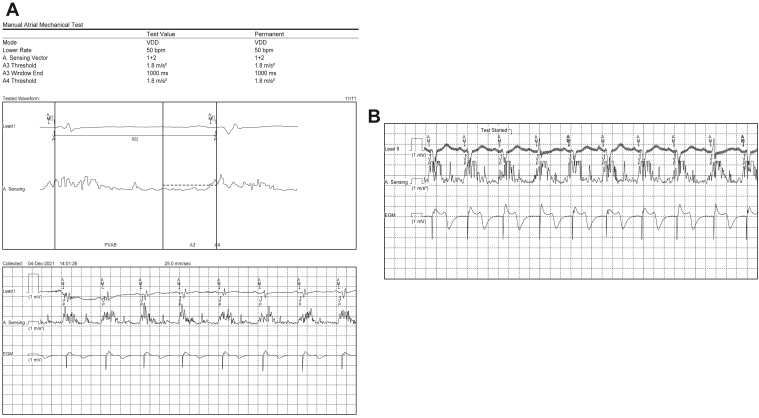
Intentional prolongation of the A3 window. **A:** Pacemaker setting and manual atrial mechanical test after intentional prolongation of the A3 window. The A3 window end was intentionally prolonged to 1000 ms. Although no VE marker was observed, the atrial signal was correctly sensed (AM) and atrioventricular (AV) synchronous pacing could be performed. **B:** The surface electrocardiogram (lead II), mechanical sensing signal, and intracardiac cardiac recording (EGM) during isoprenaline injection. Isoprenaline (2 μg) was injected to confirm atrial sensing during a high heart rate after prolongation of the A3 window in this patient. AV synchronous pacing was correctly performed after atrial mechanical sensing within the A3 windows even at a high heart rate (95 beats/min), suggesting A4 sensing within the A3 window. AM = atrial mechanical signal; EGM = intracardiac electrogram.

This patient showed a small A3 signal and a large A4 signal (Figure 2A). After confirming that the A3 threshold was adequately lower than the A4 signal, we decided to prolong the A3 window end to 1000 ms for stable atrial signal sensing. Figure 2A shows that there was no VE marker, indicating that the A4 signal was sensed in the A3 window at the baseline (heart rate 70 bpm). After the administration of isoprenaline, the A7 signal (fusion signal of A3 and A4) was sensed without the VE marker in the A3 window at a heart rate of 95 bpm (Figure 2B). After confirmation of this setting of the device, ambulatory recording during 2 days of hospitalization showed a high percentage of AV synchrony (91.3%) in a physiological histogram. The device demonstrated a high percentage of AV synchrony, 87.9%, during 4 months after the implantation.

## Discussion

We report here a novel and simplified method for achieving sufficient atrial mechanical sensing with leadless AV synchronous pacing.

Previous study showed that high AV synchrony could be predicted by the mitral flow velocity. Garweg and colleagues^6^ demonstrated E/A <0.94 with less sinus rate variability achieved high AV synchrony. They also mentioned the importance of A4 amplitude. Among the patients with persistent third-degree AV block and normal sinus rhythm, mean A4 amplitude was higher in patients with ≥90% AV synchrony than those with <90% AV synchrony.

**Figure 3:**
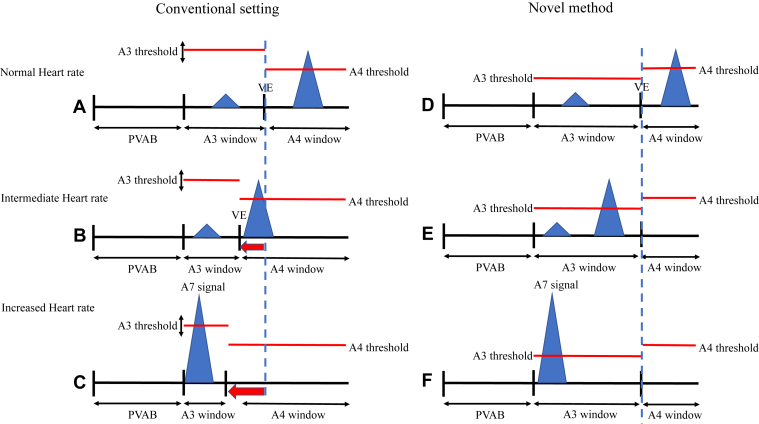
Schema of the comparison of pacemaker settings and atrial mechanical sensing between the normal setting and our novel method. **A–C:** Conventional setting of atrial mechanical sensing with the Micra AV (Medtronic, Inc, Minneapolis, MN). At a normal heart rate, small A3 (= ventricular filling) and large A4 signals (= active atrial contraction) are observed. The A3 signal is not sensed by a higher A3 threshold, although the A4 signal is correctly sensed. At an intermediate heart rate, the interval between A3 and A4 decreases. The A3 window is automatically shortened and the A4 signal can be sensed within the advanced A4 window. A3 and A4 signals fuse (and this fusion is called the A7 signal) at the higher heart rate. This duration of the A3 window is automatically changed in 15-ms steps based on observation of 8 consecutive beats. Therefore, there is a slight delay in adaptation of sensing of the atrial signal. **D–F:** Simplified A3 method by prolonging the A3 window setting. The atrial signal is promptly sensed by intentional prolongation of the A3 window irrespective of heart rate. There is no delay in sensing of the atrial signal.

In patients with a higher A4 signal and lower A3 signal than the A3 threshold, manual prolongation of the A3 window enables atrial mechanical sensing within A3 windows regardless of the change in heart rate (Figure 3D–3F). The auto A3 threshold should be turned off to avoid increasing the A3 threshold, since increasing the A3 threshold might lead to the A7 and A4 signals in the A3 window being undersensed owing to the increased A3 threshold.

The algorithm to determine A3 windows and A3 threshold is complicated and varies according to the heart rate in order to achieve adequate atrial mechanical sensing (Figure 3A–3C). This algorithm is automatically performed and cannot be recognized from the device data. At higher heart rates, the A3 windows are automatically shortened to sense the A7 signal, which is the merged signal of A3 and A4 (Figure 3C). However, this sensing algorithm still has a limitation. This automatic change of the A3 algorithm can only be applied after an 8-beat evaluation of heart rate, while the A3 window is shortened every 15 ms. This delay in change does not allow adaptation to patients with higher activity levels, resulting in poor AV synchrony during rapid changes in heart rate. Our method can maintain high AV synchrony regardless of the heart rate.

Our method has several limitations. First, it can only be adopted when the A4 signal is adequately larger than the A3 signal. Second, patients with acute heart failure are not ideal candidates for this method owing to reduced A4 signals.^6^ Oversensing of increased A3 signal might have potential risk of ventricular pacing before atrial contraction.

## Conclusion

We report a novel and simplified method, “Simplified A3 method,” for acquiring high AV synchrony regardless of heart rate. This method might facilitate better AV synchrony and quality of life in patients with LPMs.

**Key Teaching Points**

• Achievement of high atrioventricular (AV) synchrony after Micra AV (Medtronic, Inc, Minneapolis, MN) implantation is challenging at higher heart rates.
• The algorithm to determine A3 windows and the A3 threshold to achieve adequate atrial mechanical sensing is complicated and varies according to heart rate.
• In patients with higher A4 and lower A3 signals, the “Simplified A3 method” can lead to high AV synchrony regardless of heart rate.
